# Association between the ratio of total cholesterol to high-density lipoprotein cholesterol and high cardiovascular risk: A cross-sectional study

**DOI:** 10.1097/MD.0000000000048659

**Published:** 2026-05-08

**Authors:** Shuaifang Yuan, Jirui Cai, Bing He, Qiaotao Xie, Baocang Lei, Jing Bai, Nan Wang, Dongliang Liu, Qichao Wang, Jianwei Xiong, Jin Wang, Haoran Wang

**Affiliations:** a Luohe Central Hospital, Luohe Medical College, Luohe, China; b The Second Affiliated Hospital of Luohe Medical College, Luohe, China.

**Keywords:** cardiovascular disease, ChinaHEART, high cardiovascular risk, lipid ratio, total cholesterol to high-density lipoprotein cholesterol ratio, World Health Organization CVD risk chart

## Abstract

Early identification of high-risk individuals for cardiovascular disease (CVD) and effective intervention and management can significantly reduce the incidence of CVD. The ratio of total cholesterol to high-density lipoprotein cholesterol (TC/HDL-C) reflects both the protective effect of high HDL-C levels and the detrimental impact of high low-density lipoprotein cholesterol levels and residual cholesterol. However, whether the TC/HDL-C ratio can effectively identify the high CVD risk population has not been fully verified. We conducted a cross-sectional analysis using community screening data from the ChinaHEART program in Luohe, China (2021–2022). Among 6860 adults aged 35 to 75 years, 6586 with valid lipid measurements were included in the final analyses. We evaluated the association between the TC/HDL-C ratio and World Health Organization-chart–defined high 10-year CVD risk (≥20%). According to restricted cubic spline analysis, the odds of high CVD risk were increased in individuals with a higher level of TC/HDL-C. Receiver operating characteristic analysis indicated that the TC/HDL-C ratio had good discriminatory power for high CVD risk, with an area under the curve of 0.847 in the fully adjusted model. Logistic regression analysis showed that for each 1-unit increase in the TC/HDL-C ratio, the odds ratio for high CVD risk was 1.97 (95% confidence interval: 1.83–2.13, *P* < .001). Compared with the lowest quartile of TC/HDL-C, the odds ratio for high CVD risk in the highest quartile was 2.95 (95% confidence interval: 2.42–3.60, *P* < .001). This study demonstrated that the TC/HDL-C ratio was independently linked to high CVD risk. These findings highlighted the importance of considering the TC/HDL-C ratio as a valuable tool in assessing CVD risk and guiding preventive strategies.

## 1. Introduction

Cardiovascular disease (CVD) is a leading cause of global mortality and disability, imposing a heavy public health burden.^[1]^ Early identification of high CVD risk individuals and timely intervention can significantly reduce adverse CVD events, with tools such as the World Health Organization (WHO) CVD risk assessment criteria aiding in this process.^[2,3]^ Lowering low-density lipoprotein cholesterol (LDL-C) is key for primary CVD prevention, yet residual risk remains, highlighting the need for more comprehensive lipid parameters.^[4,5]^ The total cholesterol to high-density lipoprotein cholesterol (TC/HDL-C) ratio integrates protective HDL-C effects, harmful LDL-C impacts, and residual cholesterol risk, unlike single lipid parameters (e.g., TC, LDL-C) with limited predictive value, and is easy to calculate via routine tests.^[6–11]^ Despite growing evidence of its prognostic role, few studies verify its ability to identify high CVD risk individuals from general communities. We therefore hypothesize that a higher TC/HDL-C ratio is independently associated with increased CVD risk in community-dwelling adults. The current study used a community-based cross-sectional survey to investigate this association and to validate TC/HDL-C as a practical biomarker for early CVD risk stratification.

## 2. Methods

### 2.1. Study population

This cross-sectional study, conducted in compliance with the Strengthening the Reporting of Observational Studies in Epidemiology statement,^[12]^ is a sub-study of the ChinaHEART cohort, a government-supported public health initiative focused on identifying CVD risk and implementing targeted community interventions nationwide.^[13,14]^ This study was conducted among 6860 community residents in central China, recruited from 2 township hospitals in Luohe, Henan Province, between March 2021 and February 2022. The inclusion criteria encompassed the following 3 points: aged between 35 and 75 years, that is, those born between January 1, 1946, and December 31, 1986; are permanent residents of the project site, namely, those who have resided at the project site for more than 6 months out of the 12 months preceding the study; willing participants who have voluntarily signed an informed consent form. Sample size calculation was performed using the “pwr” package in R version 4.4.2 (R Foundation for Statistical Computing, Vienna, Austria), based on the primary objective of detecting an association between TC/HDL-C ratio and high CVD risk. The minimum required sample size was estimated based on the primary objective of detecting an association between TC/HDL-C ratio and high CVD risk. Assuming a significant odds ratio (OR) of 1.5 for the highest quartile of the TC/HDL-C ratio compared with the lowest quartile (reference), a two-tailed significance level (α) of 0.05, and a statistical power of 80%, the sample size was calculated. For this 2-proportion test, the baseline prevalence of high CVD risk in the lowest TC/HDL-C quartile (our reference group) was assumed to be 10% (p1 = 0.10), with an OR of 1.5. Using these parameters, the calculated minimum total sample size required was 3624 individuals (906 per quartile). The actual sample size in this study has exceeded the estimated minimum, ensuring sufficient statistical power to detect the hypothesized association. A professional research team undertook the recruitment process to ensure the generalizability and representativeness of the study population. Ethical guidelines were strictly followed throughout the study. The study protocol received approval from the institutional ethics committee of Fuwai Hospital (2014–574) and was filed with the local ethics committee of Luohe Central Hospital. Prior to participation, all individuals were provided with comprehensive information regarding the study’s purpose, significance, and associated risks. Informed consent was obtained from each participant, ensuring compliance with the relevant provisions of the Declaration of Helsinki.

### 2.2. Data collection

Data collection primarily relied on face-to-face interviews conducted by trained professionals with the study participants. The interviews encompassed a range of demographic information, such as age and gender, which aided in understanding the baseline characteristics of the study population. Data on smoking and drinking habits, comorbidities, medical history, and current medication usage were also extensively collected. We followed standardized procedures for measuring height and weight. Body mass index (BMI) was calculated as weight in kilograms divided by the square of height in meters. Additionally, we used electronic sphygmomanometers to measure blood pressure in the study participants. After a 5-minute rest in a seated position with the right arm supported, 2 readings of systolic blood pressure and diastolic blood pressure were recorded. Fasting blood glucose and lipid profiles were also assessed through fingertip blood sampling. Through this comprehensive data collection, we aimed to obtain a comprehensive understanding of the participants’ health status, serving as the foundation for subsequent data analysis and research. Of the 6860 enrolled participants, all completed the required questionnaires and blood pressure measurements as per the study protocol, ensuring no missing data for these variables. Fingertip blood glucose tests were repeated immediately if initial attempts failed, resulting in complete glucose data. However, 274 participants (4.0%) were excluded due to irreversible missing lipid profile data (invalid fingertip blood samples that could not be rectified), leaving 6586 participants for final analysis. Other covariates had minimal missingness; however, after excluding participants with invalid lipid profiles, there were no missing values in the variables presented in Table 1; therefore, the complete-case approach retained n = 6586. Using the CVD risk assessment prediction chart from the WHO guidelines for the assessment and management of CVD risk, all screened individuals underwent CVD risk assessment.^[3]^ The risk factors used to assess the CVD risk include gender, age, systolic blood pressure, TC, diabetes, and smoking history. The WHO charts we used have been calibrated by a Chinese cohort study; therefore, it is more suitable for the current study. Those with a 10-year CVD risk of ≥20% were classified as high CVD risk individuals.

**Table 1:** Baseline characteristics by TC/HDL quartiles.

Variable	Overall N = 6,586	Q1 (0.35–2.75) N = 1,647	Q2 (2.75–3.28) N = 1,647	Q3 (3.28–3.94) N = 1,646	Q4 (3.94–14.29) N = 1,646	*P*-value
Age (yr)	58 (51, 66)	59 (50, 67)	58 (50, 67)	58 (51, 66)	58 (52, 66)	.781
Gender						.097
Male	2495 (38%)	618 (38%)	612 (37%)	600 (36%)	665 (40%)	
Female	4091 (62%)	1029 (62%)	1035 (63%)	1046 (64%)	981 (60%)	
Current smoking	1320 (20%)	338 (21%)	325 (20%)	300 (18%)	357 (22%)	.089
Alcohol consumption	359 (5.5%)	86 (5.2%)	68 (4.1%)	98 (6.0%)	107 (6.5%)	.018
Systolic blood pressure (mm Hg)	137 (126, 150)	135 (124, 149)	136 (125, 150)	136 (126, 150)	142 (130, 152)	<.001
Diastolic blood pressure (mm Hg)	83 (77, 91)	83 (76, 90)	83 (76, 90)	83 (76, 90)	85 (77, 93)	<.001
Waist circumference (cm)	86 (80, 92)	83 (77, 90)	85 (79, 91)	86 (81, 93)	89 (82, 95)	<.001
Body mass index (kg/m^2^)	25.0 (23.0, 27.3)	24.1 (22.1, 26.3)	24.8 (22.7, 26.8)	25.1 (23.2, 27.4)	26.0 (24.0, 28.4)	<.001
Total cholesterol (mmol/L)	4.80 (4.11, 5.49)	4.08 (3.47, 4.65)	4.53 (4.04, 5.12)	4.97 (4.36, 5.44)	5.62 (4.98, 6.39)	<.001
High-density lipoprotein cholesterol (mmol/L)	1.43 (1.23, 1.66)	1.71 (1.49, 1.97)	1.50 (1.33, 1.69)	1.39 (1.23, 1.52)	1.21 (1.09, 1.37)	<.001
Low-density lipoprotein cholesterol (mmol/L)	2.63 (2.07, 3.15)	1.96 (1.45, 2.44)	2.48 (2.08, 2.88)	2.71 (2.33, 3.17)	3.35 (2.71, 4.03)	<.001
Triglycerides (mmol/L)	1.51 (1.14, 2.00)	1.21 (0.95, 1.62)	1.41 (1.11, 1.73)	1.58 (1.26, 2.00)	1.94 (1.47, 2.69)	<.001
Fasting glucose (mmol/L)	5.40 (5.19, 6.00)	5.40 (5.10, 5.80)	5.40 (5.10, 5.90)	5.40 (5.11, 6.00)	5.70 (5.30, 6.40)	<.001
Hypertension	1503 (23%)	334 (20%)	337 (20%)	367 (22%)	465 (28%)	<.001
Diabetes mellitus	406 (6.2%)	72 (4.4%)	96 (5.8%)	92 (5.6%)	146 (8.9%)	<.001
Coronary heart disease	93 (1.4%)	36 (2.2%)	25 (1.5%)	19 (1.2%)	13 (0.8%)	.006
Stroke	181 (2.7%)	63 (3.8%)	37 (2.2%)	41 (2.5%)	40 (2.4%)	.021
CVD high-risk status	1414 (21%)	256 (16%)	249 (15%)	290 (18%)	619 (38%)	<.001
Antihypertensive medication	861 (13%)	192 (12%)	205 (12%)	218 (13%)	246 (15%)	.035
Antidiabetic medication	252 (3.8%)	52 (3.2%)	68 (4.1%)	51 (3.1%)	81 (4.9%)	.017
Antiplatelet use	93 (1.4%)	37 (2.2%)	24 (1.5%)	20 (1.2%)	12 (0.7%)	.003
Lipid-lowering medication	248 (3.8%)	89 (5.4%)	62 (3.8%)	53 (3.2%)	44 (2.7%)	<.001

Complete-case analysis: rows with missing values in included variables were removed. Continuous variables: median (Q1, Q3). Binary variables shown as n (%). Empirical 25/50/75th percentiles: 2.7520/3.2788/3.9363. Comparisons between different groups were conducted using the Kruskal–Wallis rank sum test for continuous variables. Categorical variables were compared using the chi-square test.

CVD = cardiovascular disease, HDL = high-density lipoprotein, TC = total cholesterol.

### 2.3. Statistics

To assess the normality of continuous variables, we employed the Kolmogorov–Smirnov test. For normally distributed data, we described the distribution characteristics using the mean ± standard deviation. When the data did not follow a normal distribution, we utilized the median to represent the distribution. Additionally, we presented the distribution of continuous data based on the quartiles of the TC/HDL-C ratio. Quartiles divide the dataset into 4 equally frequent or equidistant sections, each containing about 25% of the data points. This division is concise and balanced, making it easy to understand and explain. Comparisons between different groups were conducted using the analysis of variance for normally distributed data, and the rank sum test for non-normally distributed data. Categorical variables were compared using the chi-square test, and the distribution difference between the TC/HDL-C ratio was also assessed.

To investigate the association between the TC/HDL-C ratio and high CVD risk, we performed logistic regression analysis. Three models were constructed: model 1 involved univariate logistic regression analysis without adjusting for confounders; model 2 included adjustments for age and gender; model 3 included adjustments for all the available potential confounding factors except lipid profiles, including gender, age, marital status, BMI, waist circumference, fasting blood glucose, smoking history, alcoholism, diabetes, and history of hypertension (HTN).

Furthermore, we explored potential nonlinear associations between the TC/HDL-C ratio and high CVD risk using restricted cubic spline analysis. Five key points were selected at the 10th, 25th, 50th, 75th, and 90th percentiles of the TC/HDL-C ratio.

To assess the ability of the TC/HDL-C ratio to identify high CVD risk, we employed various tools, including receiver operating characteristic (ROC) curve analysis, comprehensive discrimination improvement index, and net reclassification improvement index. ROC analyses were conducted using 3 nested logistic models consistent with those used in regression analyses: model 1 included TC/HDL-C only; model 2 added age and gender; model 3 additionally included marital status, BMI, waist circumference, fasting glucose, smoking status, alcohol use, diabetes, and HTN.

To further contextualize the performance of lipid ratios in identifying WHO-defined high CVD risk, we additionally evaluated the discriminative ability of the TC/HDL-C ratio alongside the triglycerides (TG)/HDL-C ratio. ROC curves were constructed in the same study population, with area under the curves (AUCs) estimated using bootstrap resampling to obtain 95% confidence intervals (CIs) and uncertainty bands. Differences in AUCs between the 2 ratios were assessed using the DeLong test for correlated ROC curves, and the AUC difference (ΔAUC = AUC [TC/HDL-C] − AUC [TG/HDL-C]) with a bootstrap-based CI (2000 resamples) was also reported.

**All statistical analyses were performed using Stata/BE 18.0 (StataCorp LLC, College Station ) and R version 4.4.2 (R Foundation for Statistical Computing, Vienna, Austria).**

## 3. Results

### 3.1. Study population and baseline characteristics

**Figure 1.**
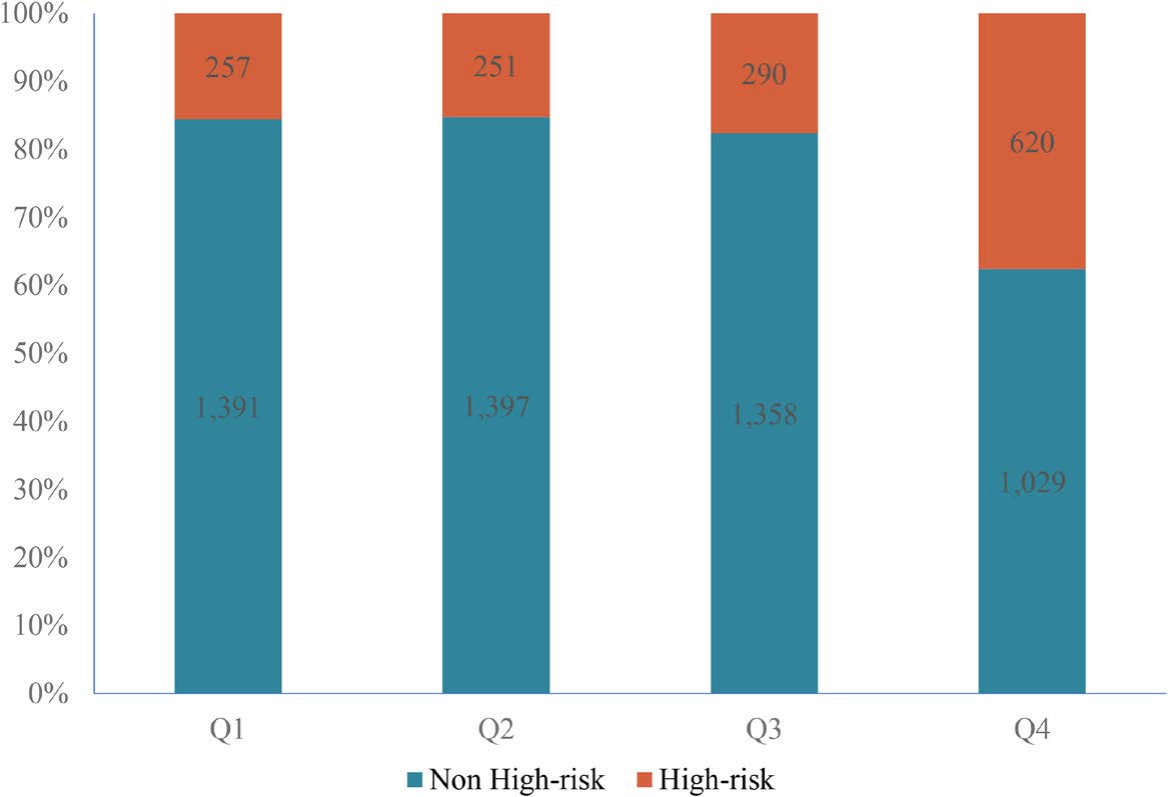
Percentage stacked histogram to illustrate the distribution of high CVD risk according to quartiles of TC/HDL-C ratio. CVD = cardiovascular disease, TC/HDL-C = total cholesterol to high-density lipoprotein cholesterol.

Of the 6860 initially enrolled community-dwelling adults, 274 (4.0%) were excluded due to invalid lipid profile data, leaving 6586 participants for analysis. Baseline comparisons in Table 1 were performed, yielding 6586 participants (1414 with high CVD risk and 5172 with non-high risk). Key trends included significant increases in metabolic/anthropometric parameters (BMI, waist circumference, blood pressure, fasting blood glucose) and higher prevalence of HTN and diabetes across increasing quartiles (all *P* < .001), while alcohol consumption (*P* = .018) showed modest but statistically significant differences. Notably, the proportion of high CVD risk rose from 16% in Q1 to 38% in Q4 (Fig. 1), suggesting a potential positive association.

### 3.2. Nonlinear association between TC/HDL-C ratio and high CVD risk (restricted cubic spline analysis)

**Figure 2.**
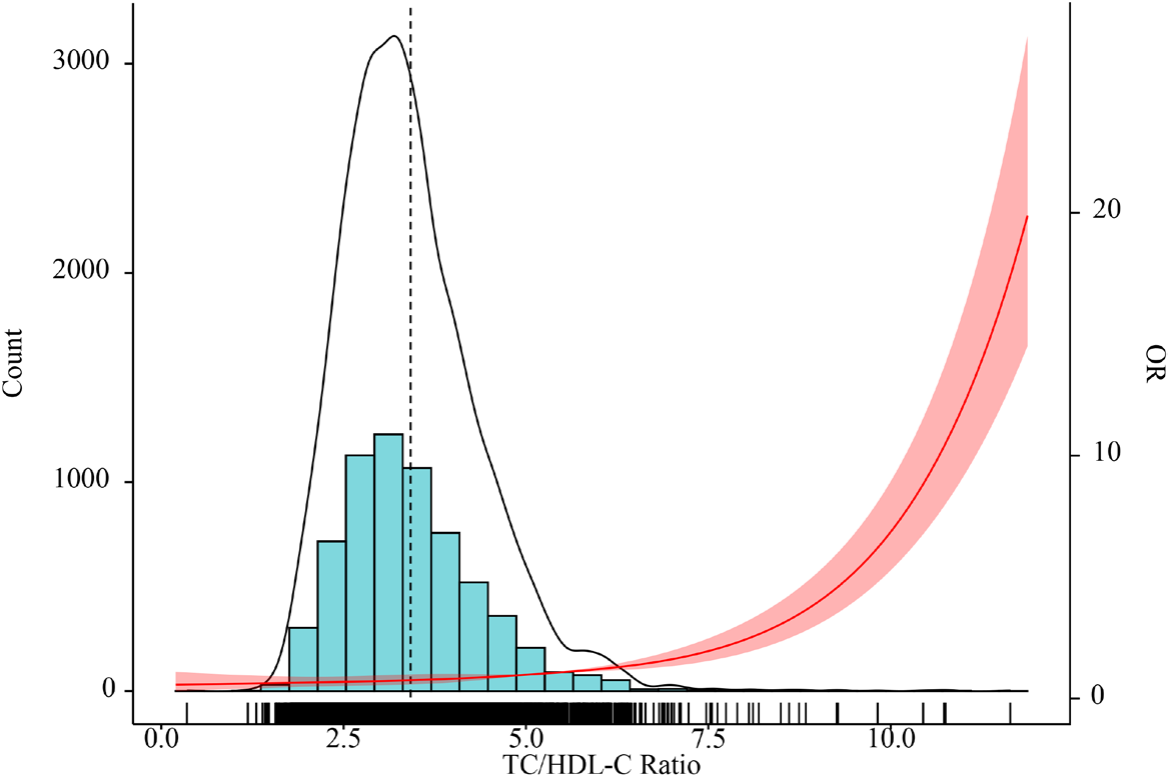
Distribution histogram of TC/HDL-C ratio in the study population and restricted cubic spline to assess the relationship between TC/HDL-C ratio and high CVD risk. For the distribution histogram, the black line indicates the distribution density. For the restricted cubic spline, the odds ratio is indicated by red solid line, and 95% CI by a shaded area. CI = confidence interval, CVD = cardiovascular disease, OR = odds ratio, TC/HDL-C = total cholesterol to high-density lipoprotein cholesterol.

Restricted cubic spline analysis (adjusted for model 3 covariates) explored the dose-response relationship, showing that high CVD risk odds increased continuously with higher TC/HDL-C ratios, with no threshold effect (*P* for nonlinearity = 0.21; Fig. 2), indicating a consistent positive association.

### 3.3. Discriminatory power of TC/HDL-C ratio for high CVD risk (ROC analysis)

**Figure 3.**
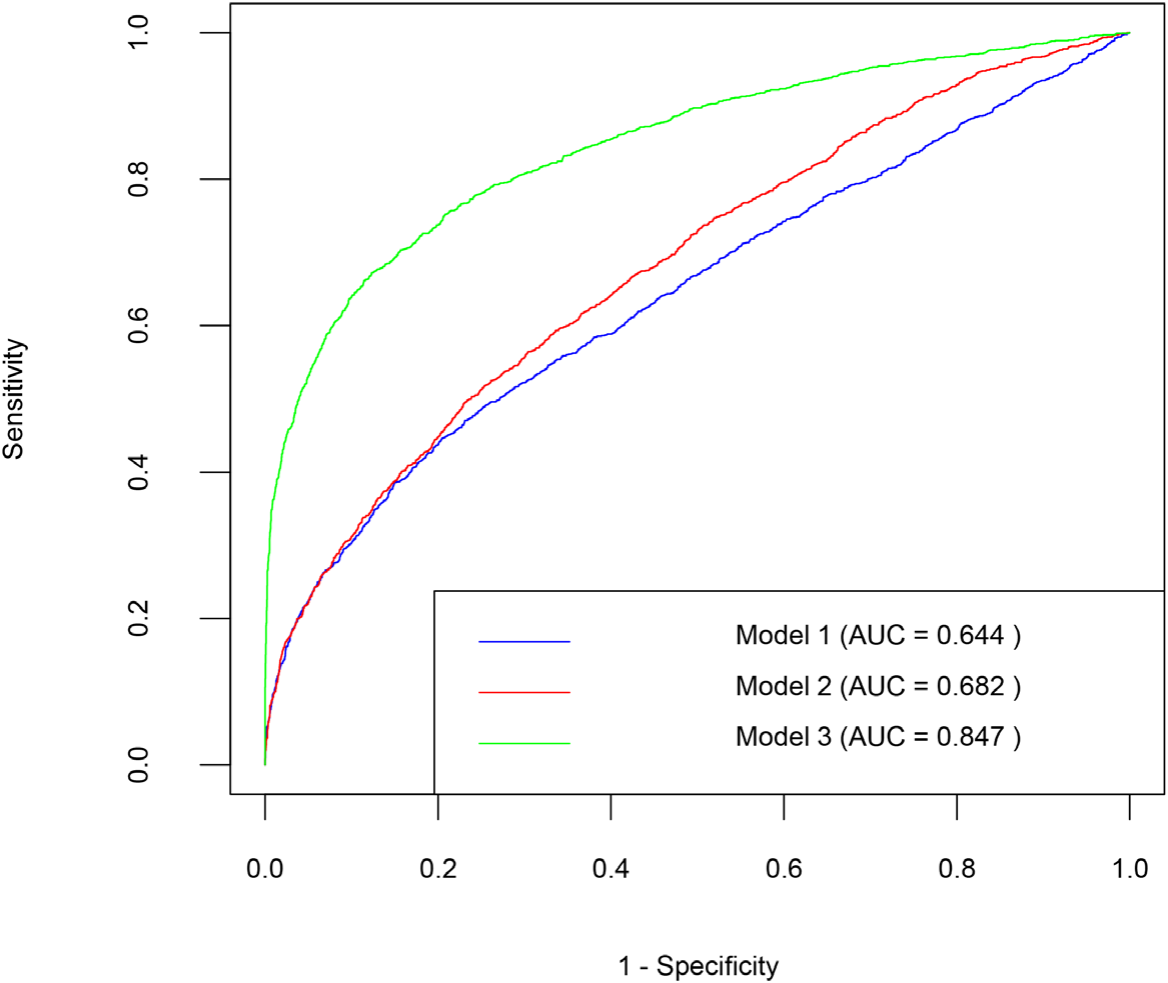
ROC curve to illustrate the discriminatory power of TC/HDL-C for high CVD risk. Model 1, no adjustment; model 2, adjusted for age and gender; model 3, adjusted for age, gender, marriage, BMI, waist, fasting glucose, smoking, alcoholic use, diabetes, and hypertension. AUC = area under the curve, BMI = body mass index, CVD = cardiovascular disease, ROC = receiver operating characteristic, TC/HDL-C = total cholesterol to high-density lipoprotein cholesterol.

ROC curve analysis (Fig. 3) showed the TC/HDL-C ratio had moderate discriminatory power alone (AUC = 0.645, model 1), improved with age and gender adjustment (AUC = 0.682, model 2), and in the fully adjusted multivariable model incorporating TC/HDL-C, age, gender, marriage, BMI, waist circumference, fasting glucose, current smoking, alcoholic use, diabetes, and HTN, the AUC was 0.847 (model 3). These results confirm enhanced identification of high CVD risk when combining the ratio with traditional factors.

### 3.4. Head-to-head ROC comparison between TC/HDL-C and TG/HDL-C

**Figure 4.**
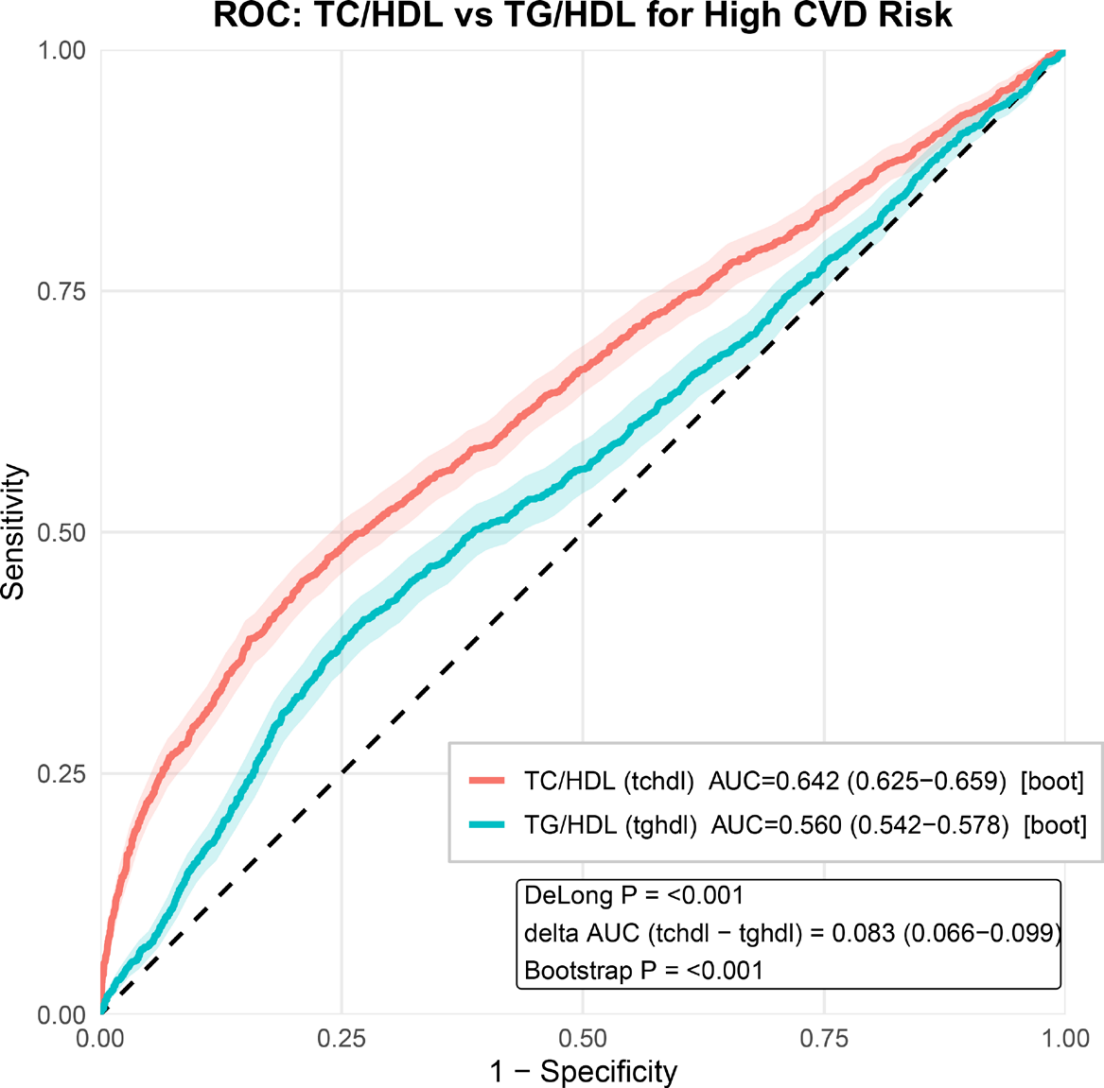
ROC comparison between TC/HDL-C and TG/HDL-C for identifying high CVD risk. ROC curves were constructed using TC/HDL-C and TG/HDL-C as single predictors. Shaded areas represent bootstrap-derived uncertainty bands. AUCs and 95% CIs were obtained by bootstrap resampling. The DeLong test was used to compare correlated AUCs, and ΔAUC (TC/HDL-C − TG/HDL-C) with bootstrap-based CI and *P*-value are shown. AUC = area under the curve, CI = confidence interval, CVD = cardiovascular disease, ROC = receiver operating characteristic, TC/HDL-C = total cholesterol to high-density lipoprotein cholesterol, TG = Triglycerides.

In the head-to-head ROC analysis, the TC/HDL-C ratio demonstrated a significantly higher discriminatory ability for high CVD risk than the TG/HDL-C ratio. The bootstrapped AUC for TC/HDL-C was 0.642 (95% CI: 0.625–0.659), compared with 0.560 (95% CI: 0.542–0.578) for TG/HDL-C (Fig. 4). The difference in AUC was ΔAUC = 0.083 (95% CI: 0.066–0.099), and the superiority of TC/HDL-C over TG/HDL-C was confirmed by DeLong test (*P* < .001) as well as bootstrap comparison (*P* < .001).

### 3.5. Association between TC/HDL-C ratio and high CVD risk (logistic regression analysis)

Logistic regression results (Table 2) showed consistent positive associations across models: per 1-unit increase in TC/HDL-C ratio, the odds of high CVD risk were 1.89 (model 1), 1.92 (model 2), and 1.97 (model 3; all *P* < .001). For quartiles (Q1 as reference), Q4 was significantly associated with higher risk (OR = 3.26 in model 1, 3.36 in model 2, 2.95 in model 3; all *P* < .001), while Q2 and Q3 showed no significance. These confirm the ratio is independently associated with high CVD risk, particularly in the highest quartile.

**Table 2:** Association between the ratio of total cholesterol to high-density lipoprotein cholesterol and high cardiovascular risk by logistic regression.

All participants	Model 1		Model 2		Model 3	
	OR (95% CI)	*P*	OR (95% CI)	*P*	OR (95% CI)	*P*
TC/HDL-C (per 1 unit)	1.89 (1.77–2.01)	<.001	1.92 (1.80–2.04)	<.001	1.97 (1.83–2.13)	<.001
TC/HDL-C (quartile)						
Q1	Ref		Ref		Ref	
Q2	0.97 (0.80–1.17)	.772	0.99 (0.82–1.20)	.916	0.89 (0.70–1.12)	.327
Q3	1.16 (0.96–1.39)	.123	1.19 (0.98–1.43)	.072	1.05 (0.83–1.32)	.699
Q4	3.26 (2.76–3.85)	<.001	3.36 (2.84–3.97)	<.001	2.95 (2.42–3.60)	<.001
Female						
TC/HDL-C (per 1 unit)	2.02 (1.86–2.19)	<.001	2.03 (1.87–2.21)	<.001	2.01 (1.84–2.19)	<.001
TC/HDL-C (quartile)						
Q1	Ref		Ref		Ref	
Q2	0.96 (0.74–1.24)	.756	0.93 (0.72–1.21)	.598	0.93 (0.71–1.20)	.564
Q3	1.40 (1.10–1.78)	.006	1.40 (1.10–1.78)	.007	1.37 (1.07–1.76)	.012
Q4	4.16 (3.34–5.19)	<.001	4.14 (3.31–5.17)	<.001	3.93 (3.11–4.96)	<.001
Male						
TC/HDL-C (per 1 unit)	1.56 (1.42–1.70)	<.001	1.60 (1.46–1.76)	<.001	1.49 (1.35–1.65)	<.001
TC/HDL-C (quartile)						
Q1	Ref		Ref		Ref	
Q2	0.96 (0.73–1.27)	.768	1.00 (0.76–1.33)	.975	1.04 (0.77–1.40)	.799
Q3	0.95 (0.72–1.25)	.691	0.98 (0.74–1.30)	.884	0.88 (0.65–1.19)	.399
Q4	2.18 (1.70–2.79)	<.001	2.35 (1.83–3.03)	<.001	1.94 (1.47–2.58)	<.001

Model 1, no adjustment; model 2, adjusted for age and gender; model 3, adjusted for age, gender, marriage, BMI, waist, fasting glucose, smoking, alcoholic use, diabetes, and hypertension.

BMI = body mass index, CI = confidence interval, OR = odds ratio, TC/HDL-C = the ratio of total cholesterol to high-density lipoprotein cholesterol.

## 4. Discussion

In our study, we observed a significant positive correlation between the TC/HDL-C ratio and high CVD risk, even after adjusting for relevant risk factors. The use of ROC analysis indicated that when the TC/HDL-C ratio was combined with age, gender, personal medical history, history of HTN/diabetes, and blood pressure and glucose levels, it provided superior discrimination in identifying individuals at high risk for CVD. Notably, the TC/HDL-C ratio is derived from routine examinations, making it a practical and effective tool for assessing CVD risk.

We previously reported the association between the TG/HDL-C ratio and cardiovascular high risk in a separate cross-sectional study.^[15]^ In the current work, our focus is distinct – evaluating the TC/HDL-C ratio as the primary exposure and characterizing its association and risk stratification utility within the same community-screened population using WHO-calibrated CVD risk charts. To enhance transparency and address potential concerns about overlap, we conducted a prespecified head-to-head comparison between TC/HDL-C and TG/HDL-C. In this analysis, TC/HDL-C showed significantly better discrimination than TG/HDL-C for identifying high CVD risk (higher AUC and significant ΔAUC by both DeLong and bootstrap testing; Fig. 4), supporting that TC/HDL-C captures complementary information beyond TG/HDL-C in this setting.

From a pathophysiological perspective, TG/HDL-C is often considered a marker related to insulin resistance and atherogenic dyslipidemia, whereas TC/HDL-C more directly reflects the balance between the overall atherogenic cholesterol burden and protective HDL-C. This may explain why TC/HDL-C provided superior discrimination for WHO-defined high CVD risk in our cohort, where TC is itself a key component of the risk chart.

Dyslipidemia is a necessary condition for the development of atherosclerosis. However, it has been demonstrated that the predictive ability of any single lipid parameter for CVD is limited.^[16]^ TC encompasses not only LDL-C and HDL-C measured in routine testing, but also residual cholesterol from triglyceride-rich lipoprotein remnants, including chylomicron remnants, very-low-density lipoprotein, and intermediate-density lipoprotein.^[17]^ The TC/HDL-C ratio reflects both the protective effect of high HDL-C levels on CVD and the detrimental impact of high LDL-C levels, as well as, to some extent, the CVD risk associated with residual cholesterol.^[18]^ Furthermore, the TC/HDL-C ratio has the advantage of being straightforward to measure and calculate, making it potentially more suitable for widespread implementation in screening high-risk populations for CVD.

The current finding resonates with and diverges from several previous studies in the field. Wen et al found that TC/HDL-C ratio was associated with arterial stiffness even when LDL-C was below 70 mg/dL.^[11]^ Elshazly et al’s big data analysis indicates that the TC/HDL-C ratio could provide additional information to LDL-C and non-HDL-C, highlighting the potential of this ratio in refining CVD risk assessment.^[19]^ Manubolu et al found the TC/HDL-C ratio to be an independent associate of noncalcified plaques, suggesting its involvement in atherosclerotic plaque instability.^[20]^ Tan et al also showed that the lipid accumulation product index correlates with CVD risk, supporting the view that integrating multiple lipid-related risks would offer superior predictive value over single parameters.^[21]^ Quispe et al revealed that discordance between the TC/HDL-C ratio and other lipid measures was associated with a more atherogenic clinical profile and increased CVD risk.^[22]^ As for its predictive role for CVD events, while Jung et al reported no association between the TC/HDL-C ratio and major adverse cardiac event survival rates post-percutaneous coronary intervention,^[23]^ Yu et al and Zhou et al reported nonlinear associations between the TC/HDL-C ratio and CVD hospitalization, rehospitalization, and all-cause mortality, respectively.^[8,24]^ This discrepancy may be attributed to the different study populations and endpoints considered and suggests a complex relationship between the ratio and CVD outcomes, with potential thresholds that may influence clinical decision-making. Furthermore, Elshazly et al found that the TC/HDL-C ratio, when discordant with LDL-C and other lipid parameters, reclassifies atheroma progression and major adverse cardiac events rates.^[25]^ Furthermore, Hatmi et al’s evidence supports aggressive treatment strategies targeting the TC/HDL-C ratio for attenuating the risk of myocardial infarction in younger individuals, which may have implications for our study’s findings in a broader clinical context.^[26]^ Recent studies have further supported these observations; for example, Wang et al investigated changes in the non-high-density lipoprotein to high-density lipoprotein ratio (closely related to TC/HDL-C) and its link to CVD in a population-based study.^[27]^ Yuan et al explored the relationship between lipid ratios such as LDL-C/HDL-C (alongside inflammatory markers) and coronary artery disease progression.^[28]^ Li et al found that lipid ratios such as TC/HDL-C correlate with the severity of coronary artery lesions in patients with coronary heart disease.^[29]^ Qu et al evaluated the TC/HDL-C ratio and triglyceride-glucose index for their diagnostic utility in coronary artery disease.^[30]^ Yang et al detected a J-shaped association between the non-high-density lipoprotein to high-density lipoprotein ratio and the prevalence of CVD.^[31]^ Noh et al associated higher TC/HDL-C ratios with increased mortality in incident peritoneal dialysis patients, highlighting its prognostic value in high-risk groups.^[32]^

This study has notable strengths, including a well-designed study approach and a rigorous research process management system. We collected clinical data based on relevant criteria, encompassing medical history assessment, risk factor evaluation, and measurement of blood pressure and lipids. This ensured the accuracy and completeness of our data. Additionally, we employed multivariate logistic regression and adjusted for traditional CVD risk factors, enhancing the robustness and reliability of our results. However, there are several limitations to consider. First, our study focused on a local branch of a national study, which may limit the generalizability of our findings to the broader population. Moreover, our estimation of target parameters solely relied on blood lipid measurements, potentially overlooking the influence of population, regional disparities, and lifestyle factors. Second, our study had a retrospective observational design, preventing us from evaluating the predictive value of the TC/HDL-C ratio for future CVD adverse events or determining whether interventions targeting this ratio could reduce the incidence of subsequent CVD events. To address these questions, continued follow-up of the study population is necessary.

In summary, our study revealed a notable increase in the prevalence of high CVD risk among individuals with elevated TC/HDL-C ratios compared to those with lower ratios. These findings suggest that the TC/HDL-C ratio could serve as an independent risk factor for CVD risk, with higher ratios indicating heightened risk. Considering its ease of calculation from standard laboratory results, the TC/HDL-C ratio could be a practical and cost-effective adjunct for clinicians. In primary care settings, where resources are often limited, this simple metric may serve as an initial screening tool to help identify individuals who warrant more comprehensive evaluation. For those found to have a high ratio, it could prompt earlier and more aggressive management of modifiable risk factors, such as recommending dietary modifications, increasing physical activity, or initiating lipid-lowering therapy when appropriate. Given the cross-sectional nature of our study, these interpretations should be considered hypothesis-generating. Future prospective studies and randomized controlled trials are needed to validate whether using the TC/HDL-C ratio in clinical practice leads to improved long-term CVD outcomes and reduced event rates. Ultimately, the application of this simple ratio has the potential to enhance early risk stratification, thereby informing targeted preventive strategies and contributing to the reduction of the overall public health burden of CVD. Because TC is an input variable in the WHO CVD risk charts used to define the high-risk outcome, the observed association and discrimination of TC/HDL-C may partly reflect structural overlap with the risk algorithm; therefore, TC/HDL-C should be interpreted as a practical lipid-derived marker for WHO-chart risk categorization rather than a wholly independent predictor outside the chart variables.

## Author contributions

**Conceptualization:** Haoran Wang.

**Funding acquisition:** Haoran Wang.

**Methodology:** Haoran Wang.

**Data curation:** Shuaifang Yuan, Bing He, Qiaotao Xie, Baocang Lei, Jing Bai, Nan Wang, Dongliang Liu, Jianwei Xiong, Haoran Wang.

**Formal analysis:** Shuaifang Yuan, Bing He, Qiaotao Xie, Jing Bai, Dongliang Liu, Jianwei Xiong, Haoran Wang.

**Investigation:** Jirui Cai, Baocang Lei, Jing Bai, Nan Wang, Dongliang Liu, Qichao Wang, Jin Wang, Haoran Wang.

**Project administration:** Haoran Wang.

**Writing – original draft:** Shuaifang Yuan, Jirui Cai, Bing He, Qiaotao Xie, Jing Bai, Nan Wang, Dongliang Liu, Qichao Wang, Jianwei Xiong, Jin Wang.

**Writing – review & editing:** Haoran Wang.
